# Gender and age differences in the association between living arrangement and physical activity levels among youth aged 9–19 years in Shanghai, China: a cross-sectional questionnaire study

**DOI:** 10.1186/s12889-019-7383-z

**Published:** 2019-08-01

**Authors:** Xiang Fan, Zheng Zhu, Jie Zhuang, Yang Liu, Yan Tang, Peijie Chen, Zhen-bo Cao

**Affiliations:** 1 Department of Physical Education, Shanghai Jiao Tong University, 800 Dong Chuan Road, Shanghai, 200240 China; 2 School of Kinesiology, Shanghai University of Sport, 399 Chang Hai Road, Shanghai, 200438 China; 3 School of Physical Education and Sport Training, Shanghai University of Sport, 399 Chang Hai Road, Shanghai, 200438 China; 4 Shanghai Research Center for Physical Fitness and Health of Children and Adolescents, Shanghai University of Sport, 399 Chang Hai Road, Shanghai, 200438 China

**Keywords:** Questionnaire, Daily activity, Family, Children, Adolescent, Youth

## Abstract

**Background:**

We examined the correlations between living arrangement and the physical activity (PA) levels of youth aged 9–19 years while accounting for demographic factors such as age, sex, and socioeconomic status in Shanghai, China.

**Methods:**

Cross-sectional analyses of data from the 2014 Physical Activity and Fitness in Shanghai China—The Youth Study was conducted. Participants were 33,213 primary (9–11-year-olds; *n* = 13,237), junior middle (12–14-year-olds; *n* = 11,157), and junior high school students (15–19-year-olds; *n* = 8819). Youth (boys = 49%) and their guardians were randomly sampled from 17 districts in Shanghai, China. Youths’ moderate-to-vigorous PA (MVPA) levels, information about living arrangement, and guardians’ sociodemographic factors were collected via questionnaires.

**Results:**

Only 17.8% of school-aged youths in Shanghai met MVPA recommendations, with significantly more boys (20.6%) meeting recommendations than girls (*p* < .001). Youths living in rural areas showed an overall significantly higher percentage of meeting MVPA recommendations (20.3%) than those living in urban areas (*p* < .001). Youths who lived with single parents showed an overall significantly lower percentage of meeting MVPA recommendations (15.3%) than those living with their grandparent(s) or with both parents (*p* < .001). A logistic regression analysis revealed that, among 9–11-year-olds, children who live with their grandparent(s) were less likely to meet MVPA recommendations than those who lived with both parents (boys: adjusted odds ratio (aOR) = 0.72, 95% confidence interval (CI) = 0.61–0.84; girls: aOR = 0.84, 95%CI = 0.72–0.98).

**Conclusions:**

Type of living arrangement was associated with the PA of youth in Shanghai, with no significant gender difference. Youth aged 9–19 years who lived with single parents had the lowest percentage of meeting MVPA recommendations. The probability of achieving 60 min/day MVPA recommendations was significantly lower among 9–11-year-old children living with their grandparent(s) than children living with both parents; however, no such difference was observed among adolescents. Our findings suggest that living arrangement may be an important consideration for promotion of PA among youth in China.

## Background

Regular physical activity (PA) is a well-documented contributor to youths’ health including aerobic fitness, blood lipids, blood pressure, body composition, glucose metabolism, skeletal health, and psychological health [1, 2]. The World Health Organization (WHO) issued a set of PA guidelines specifically for youths [3]. These guidelines recommended that youths engage in at least 60 min of daily moderate-to-vigorous PA (MVPA). However, past studies have shown problematic levels of compliance to PA recommendations. Worldwide, approximately 80% of adolescents (13–15-year-olds) do not adhere to these recommendations [4–6]. In China, only 30% of youths (9–17-year-olds) met the MVPA recommendations [7].

Exploring factors that may be related to youths’ PA behavior is important for informing strategies to intervene in these health behaviors. Previous studies showed that family has a powerful and direct influence on children’s PA [8]. Some studies showed a strong relationship between a youths’ PA and sports participation and parents’ PA: parents’ and youths’ activity levels include the parents’ serving as role models [9–12]. The potential mechanisms of family influence had been reviewed by Taylor and colleagues [13]. They found that parents can support or hinder their children’s PA in direct ways by controlling access to environments that facilitate PA and indirect ways by transporting children to sports facilities [13]. Thus, changes in family structure, especially the dominance of the conventional two-parent family declining, may have an influence on children’s PA. A study of children in South Africa reported a positive association between two parent family was positively and children’s PA [14]. In contrast, a study of children’s PA in the United States found an inverse relationship between two-parent family and boys’ PA [15]. Nonetheless, a limited number of studies and inconsistent findings imply that more research is necessary to determine the relationship between family factors and youths’ PA levels.

Meanwhile, three decades of open reforms in China has resulted in unprecedented economic and social environmental changes have made a significant impact on family structure in both rural and urban areas [16, 17]. As people become more tolerant of divorce, the number of single-parent families continues to increase [17, 18]. A study in China reported that children living in single-parent households spent more time on MVPA than did those living in two-parent homes [19]. However, the sample size of this study was relatively small (*n* = 612). In contrast, a study involving Chinese nationally representative samples reported that children living with two parents scored higher on MVPA than those from single-parent families [20]. In addition, whether urban or rural area, grandparent(s) caring for grandchildren is an increasingly common experience for many dual-income families in China [21]. Previous studies have revealed that grandparents tend to overprotect their grandchildren, which had a negative association between grandparents’ living habits and MVPA duration among 9-to-11-year-olds [19, 21]. Since the social transformation in families may be a vital source of influence on youths’ lifestyle, we hypothesized a correlation between family living arrangements and youths’ PA levels in Shanghai, China. Furthermore, previous studies found that children in urban areas had more exercise equipment available at home, and they were transported more frequently to places where they could be physically active [22]. Therefore, the confounding effects of residential location needs to be considered when investigating the associations between living arrangement and PA levels among youth.

Considering all these factors, large-scale studies are necessary to inform public policy. As part of an on-going public health effort to track and evaluate PA in school-aged children, in 2014, the PA and Fitness in Shanghai China—The Youth Study (PAFSCTYS) was conducted. Using PAFSCTYS data, we investigated the associations between diverse living arrangements and youths’ (aged 9–19 years) PA levels from both urban and rural areas in Shanghai, China, while accounting for distinct demographic factors.

## Methods

### Study design and sampling procedure

The data were acquired from the PAFSCTYS, a government survey project sponsored by the Shanghai Municipal Education Commission; this was conducted from October to December 2014. The PAFSCTYS employed a cross-sectional, multistage sampling design to survey PA level of children and adolescents in primary, junior middle, and junior high schools from the school education system in Shanghai, China.

Since students below the 4th grade were not considered capable of understanding the questionnaire, we only included 4th to 12th grade students in this study. According to this framework, the participants were recruited using a 2-stage cluster (non-probability) sampling design described below.

In the first stage, a total of 711 primary, junior middle school, and junior high schools were selected from all 17 districts of the Shanghai metropolitan area. The second stage was the selection of school grades within a sample school in which 1–2 grade classes were recruited. Among primary schools (Grades 4–6), junior middle and junior high schools, student inclusion required that each grade class consist of at least 20 students (10 boys and 10 girls). If the student quota was not met, supplementary samples would be taken from other schools in the same district.

The study was approved by the Institutional Review Board (IRB) of the Shanghai University of Sport (SUS) in 2014. Due to the fact that none of survey items related to personal ethic issue, the IRB of SUS approved that verbal consent is sufficient and written consent is waived.

### Participants

Participants were 43,416 children from primary schools (Grades 4–6, *n* = 16,752), junior middle schools (Grades 7–9, *n* = 14,545), and junior high schools (Grades 10–12, *n* = 12,119) schools, with student ranging in age from 9 to19 years old. In response, 38,988 students (response rate = 89.8%) completed the self-reporte questionnaire.

### Procedures

The verbal consent protocol approved by the IRB of SUS required that before data collection, research assistants must clarify to teachers and principals of each school about the study purposes, potential risks and benefits of the study participation, and acquire permission to conduct the study. All children and their parents or guardians involved in the study were advised specifically that participation was completely voluntary. Verbal consent was obtained from all parents or guardians, and positive assent was obtained verbally from all children before data collection. Trained research assistants implemented the survey according to a standardized survey administration protocol during prearranged regular school hours. The survey was completed either online (78%) or on paper (22%) in a classroom setting. Students were instructed on how to fill out the survey and were provided ample time for questions. Data from the survey were collected and analyzed anonymously.

### Measures

Participants’ PA level and information concerning guardians’ education and occupation levels were obtained using two questionnaires that were developed collaboratively by a multidisciplinary team of researchers based on previous literature and a pilot study to ensure consistency and reliability [23]. Details of each measure in this study are described below.

#### Physical activity

Participants were asked to report the number of days that they met the PA guidelines (≥60minMVPA/day) in the past week on the questionnaire. Those items have been confirmed as feasible and reliable measures of PA for Chinese children and adolescents [24]. To calculate MVPA, each student answered the following question: “During the past 7 days, on how many days did you engage in 60 minutes or more per day of MVPA.” MVPA was defined as any other physical activities that increase your heart rate and make you breathe hard and sweat (e.g., walking, biking to school, recreational swimming, jogging, team sports, fast dancing, and jump-rope). Responses ranged from 0 to 5 days for weekdays and from 0 to 2 for weekends. Children were categorized as meeting the recommendations if the response to the PA question was affirmative for a total of 7 days per week [25].

#### Family demographics

The questionnaire completed by guardians requested information about their age, gender, family’s residential location, living arrangement and family socioeconomic status (SES). The residential location of participants’ family included two options: urban area or rural area, which was determined by factors such as geographical location, population density and economic development level that were issued by the National Bureau of Statistics of China [26].

Living arrangement was collapsed into three categories: 1) living with both biological parents only; 2) living with one biological parent only; 3) family members include grandparent/grandparents, and they are the primary caregivers for young children. Guardians’ SES was investigated based on education and occupation [26]. The education categories were included six options: 1) lower than Grade 7; 2) junior high school level (Grade 9); 3) partial high school level (Grade 12); 4) partial college or specialized training; 5) standard college or university graduation; 6) graduate professional training (graduate degree). The occupational status was indirectly obtained by asking the participants about the type of work. We coded these types of work based on the following census categories: 1) unemployed; 2) farm laborers, menial service workers, and unskilled workers; 3) small business owners, skilled manual workers, craftsmen, and tenant farmers; 4) managers and minor professionals; 5) administrators and proprietors of medium-sized businesses; 6) high executives, proprietors of large businesses, and major professionals. The SES score of an individual was calculated by multiplying the scale value for occupation by a weight of 5 and the scale value for education by a weight of 3. Education level scores ranged from 3 to 18; occupation level scores ranged from 5 to 30; and the total social status index ranged from 8 to 48and was categorized as high (35 to 48), moderate (22 to 34), and low (8 to 21). This instrument’s validity and reliability were established by Cirino et al. [27].

#### Student characteristics

Students were asked to report their age, grade, gender. Students’ height was measured to the nearest 0.1 cm in bare feet; body weight was measured to the nearest 0.1 kg. Both measures were assessed using a portable instrument (GMCS-IV, Jianmin, Beijing, China). Participants’ body mass index (BMI) was calculated as weight in kilograms divided by the square of height in meters (kg/m^2^). The measurements were conducted by accredited fitness staff.

## Statistical analyses

All analyses were conducted using SPSS 22.0 software (SPSS Inc., Chicago, IL, USA). The missing cases and abnormal values were removed: deleting missing cases (or having abnormal values) for grade (*n* = 1311), gender (*n* = 874), PA on weekdays and weekend days (*n* = 3590). In total, 33,213 eligible cases were included in the analytical dataset. Descriptive statistics on the prevalence of meeting PA recommendations were calculated by age, sex, residence locale, guardians’ SES and living arrangement. Continuous variables are presented as means ± standard deviation (SD) and categorical variables are presented as percentages, unless otherwise indicated. Between-group differences in demographic variables were tested with a chi-square test for categorical variables. Differences in adherence to recommendations were analyzed using a logistic regression analysis with odds ratios (ORs) and 95% confidence intervals (CIs). All analyses were adjusted for children’s chorological age, BMI, residential location, and family SES. Tests were considered statistically significant at an overall α level of .05 (two-sided).

## Results

### Sample characteristics

Of the total of 38,988 school children who participated in the survey portion of the PAFCTYS, our analyses included valid data obtained from 33,213 children (16,310 boys and 16,903 girls), with 13,237 (29.4%) in Grades 4–6(9–11-years-old), 11,157 (34.7%) in Grades 7–9(12–14-years-old), and 8819 (35.9%) in Grades 10–12(15-to-19yearsold). Participants’ demographic characteristics are presented in Table 1. Participants’ mean age was 13.0 ± 2.5 year.

**Table 1:** Participants’ demographic characteristics

Variable	Overall *(n* = 33,213)			Age group, years								
				9–11 (*n* = 13,237)			12–14 (*n* = 11,157)			15–19 (*n* = 88,19)		
	Boys	Girls	Effect size	Boys	Girls	Effect size	Boys	Girls	Effect size	Boys	Girls	Effect size
Sample size (*n*)	16,310 (49.1)	16,903 (50.9)		6733 (50.9)	6504 (49.1)		5385 (48.3)	5772 (51.7)		4192 (47.5)	4627 (52.5)	
Age (years)^a^	13.3 ± 2.5	13.2 ± 2.5	n.s	10.2 ± 0.8	10.2 ± 0.8	n.s	13.0 ± 0.8	13.0 ± 0.8	n.s	16.2 ± 1.0	16.1 ± 1.0	n.s
Height (cm)^a^	151.2 ± 18.3	148.2 ± 14.9	0.27	136.8 ± 10.5	136.6 ± 11.3	0.02	161.7 ± 10.3	158.2 ± 6.9	0.40	173.3 ± 6.7	162.1 ± 5.9	1.31
Weight (kg)^a^	46.7 ± 17.8	42.3 ± 13.9	0.34	34.9 ± 10.6	32.5 ± 9.4	0.27	54.2 ± 13.8	49.7 ± 10.2	0.36	65.9 ± 13.8	55.0 ± 12.3	0.86
BMI (kg/m^2^)^a^	19.7 ± 4.2	18.7 ± 3.6	0.26	18.3 ± 3.7	17.1 ± 3.0	0.38	20.5 ± 4.0	19.8 ± 3.5	0.20	21.9 ± 4.2	20.9 ± 3.3	0.26
Residential location (%)^b,c^												
Urban	10,490 (66.9)	10,892 (66.6)	n.s	3591 (66.8)	3690 (67.9)	n.s	3790 (67.1)	3964 (67.3)	n.s	2987 (66.8)	3115 (64.3)	0.03
Rural	5187 (33.1)	5452 (33.4)		1781 (33.2)	1744 (32.1)		1862 (32.9)	1923 (32.7)		1485 (33.2)	1726 (35.7)	
SES												
Low	6988 (43.3)	7498 (44.1)	n.s	3122 (46.9)	2994 (45.5)	0.03	2285 (42.9)	2496 (43.0)	n.s	1581 (38.0)	2008 (43.5)	0.06
Moderate	4357 (27.0)	4451 (26.2)		1856 (27.9)	1759 (26.8)		1382 (26.0)	1511 (26.0)		1119 (26.9)	1181 (25.6)	
High	4793 (29.7)	5047 (29.7)		1683 (25.3)	1822 (27.7)		1654 (31.1)	1798 (31.0)		1456 (31.1)	1427 (30.9)	
Living with (%)^b^												
Both parents	13,572 (83.2)	13,862 (82.0)	0.02	5498 (81.7)	5148 (79.2)	0.04	4454 (82.7)	4761 (82.5)	n.s	3620 (86.3)	3953 (85.4)	n.s
One parent	911 (5.6)	954 (5.6)		307 (4.5)	278 (4.3)		345 (6.4)	331 (5.7)		259 (6.2)	345 (7.5)	
Grandparent(s)	1827 (11.2)	2087 (12.4)		928(13.8)	1078 (16.5)		586 (10.9)	680 (11.8)		313 (7.5)	329 (7.1)	

^a^ Data presented as mean ± standard deviations. ^b^ Data presented as n (%). ^c^ 1192 children had missing information on residence identity, ^d^ 79 children had missing information on SES. Abbreviation: *n.s.* no significance, *BMI* body mass index

### Prevalence of meeting MVPA recommendations

The estimated prevalence of youths’ adherence to current PA recommendations is shown in Table 2. In 2014, about 17.8% of children and youths in Shanghai met MVPA recommendations, with a significantly higher percentage observed for boys than for girls (15.1%), a pattern that is consistent across the three age groups (Table 2). Regardless of sex, inactivity rates increased with age. Further, youth living in rural areas had a significantly higher percentage of meeting MVPA recommendations than those living in urban areas. Youth who lived with one parent showed a significantly lower percentage of meeting MVPA recommendations than those living with their grandparent(s) or both parents.

**Table 2:** Prevalence of meeting MVPA recommendations by sex, age groups, residence locales, and living arrangement

Variable	Overall	Age group, years		
	*(n* = 33,213)	9–11 *(n* = 13,237)	12–14 (*n* = 11,157)	15–19 (*n* = 8819)
Overall (% (SE))	17.8 (0.2)	28.8 (0.3)	14.2 (0.3)	5.7 (0.2)
Sex				
Boys	20.6 (0.2)*	29.9 (0.4)^#^	18.0 (0.4)	8.8 (0.4)
Girls	15.1(0.2)	27.7 (0.4)^#^	10.7 (0.4)	3.0 (0.3)
Residence locale				
Urban				
Overall	16.4 (0.3)	26.3 (0.5)	13.4 (0.5)	5.9 (0.5)
Boys	18.9 (0.3)	27.7 (0.6)	16.8 (0.6)	8.6 (0.5)
Girls	14.0 (0.3)	24.8 (0.6)	10.3 (0.5)	3.4 (0.4)
Rural				
Overall	20.3 (0.4)^a^	33.3 (0.5)	15.9 (0.5)	5.4 (0.5)
Boys	23.7 (0.3)^a^	34.2 (0.6)	20.4 (0.5)	9.4 (0.6)
Girls	17.0 (0.3)^a^	32.4 (0.6)	11.8 (0.4)	2.2 (0.5)
SES				
Low				
Overall	19.4 (0.4)^b^	31.1 (0.5)	15.5 (0.5)	4.8 (0.6)
Boys	22.1 (0.3)^b^	32.0 (0.6)	18.7 (0.6)	7.5 (0.6)
Girls	16.7 (0.3)^b^	30.2 (0.5)	12.1 (0.6)	2.6 (0.5)
Moderate				
Overall	17.7 (0.3)	27.9 (0.6)	14.1 (0.6)	6.2 (0.5)
Boys	20.1 (0.3)	28.4 (0.6)	17.7 (0.5)	9.0 (0.5)
Girls	15.3 (0.3)	27.2 (0.6)	10.8 (0.6)	3.6 (0.6)
High				
Overall	15.6 (0.3)	25.7 (0.6)	12.9 (0.5)	6.5 (0.6)
Boys	18.8 (0.4)	27.9 (0.6)	17.4 (0.6)	10.0 (0.6)
Girls	12.5 (0.4)	23.6 (0.6)	8.8 (0.6)	3 .0 (0.6)
Living with				
Both parents				
Overall	17.9 (0.3)	29.7 (0.5)	14.3 (0.5)	5.7 (0.4)
Boys	20.8 (0.3)	30.9 (0.6)	18.0 (0.5)	8.8 (0.5)
Girls	15.1 (0.4)	28.4 (0.4)	10.9 (0.6)	2.9 (0.4)
One parent				
Overall	15.3 (0.4)^c^	26.3 (0.4)	14.5 (0.5)	5.5 (0.6)
Boys	19.9 (0.3)	29.3 (0.5)	20.0 (0.6)	8.5 (0.6)
Girls	10.9 (0.4)	23.0 (0.4)	8.8 (0.5)	3.2 (0.5)
Grandparent(s)				
Overall	18.1 (0.3)	24.9 (0.5)	13.4 (0.5)	6.4 (0.6)
Boys	19.4 (0.4)	24.5 (0.4)	17.1 (0.5)	9.6 (0.5)
Girls	16.9 (0.3)	25.2 (0.5)	10.3 (0.5)	3.3 (0.6)

^*^p < .001, compared with girls. ^#^ *p* < .001, compared with counterparts from the other two age groups

^a^*p* < .001, compared with urban counterparts. ^b^*p* < .001, compared with counterparts from the other SES

^c^*p* < .001, compared with counterparts from the other living arrangements

Abbreviations: *SE* standard error, *MVPA* moderate-to-vigorous physical activity

### Differences in adherence to MVPA recommendations

Differences in adherence to PA recommendations by demographic characteristics and living arrangement were examined using a logistic regression analysis. Overall, girls were less likely to meet MVPA recommendations compared to boys. Relative to participants aged 9–11 years, participants aged 12–14 years and 15–19 years were less likely to meet MVPA recommendations. Further, participants living in rural areas were more likely to meet the recommendations compared to children living in urban areas (Table 3). Moreover, 9-to-11 year old participants who lived with their grandparent(s) were less likely to meet MVPA recommendations than those who lived with both parents; however, no such associations between MVPA recommendations and living arrangement were observed in the other age groups (Table 4).

**Table 3:** Adjusted odds ratio (95%CI) of meeting PA recommendation by sex, age groups, residence locales, and living arrangement^a^

Variable	Physical activity			
	OR (95% CI)	*p-*value	aOR (95% CI)	*p-*value
Sex				
Boys	*Reference*		*Reference*	
Girls	0.69 (0.65–0.73)	< .001	0.68 (0.64–0.72) ^a^	< .001
Age group, years				
9–11	*Reference*		*Reference*	
12–14	0.37 (0.33–0.41)	< .001	0.45 (0.40–0.51) ^a^	< .001
15–19	0.15 (0.14–0.27)	< .001	0.22 (0.19–0.26) ^a^	< .001
Residential location				
Urban	*Reference*		*Reference*	
Rural	1.36 (1.23–1.47)	< .001	1.34 (1.26–1.43) ^a^	< .001
SES				
Low	1.30 (1.21–1.39)	< .001	1.30 (1.21–1.40) ^b^	< .001
Moderate	1.12 (1.04–1.20)	< .001	1.12 (1.04–1.21) ^b^	< .001
High	*Reference*		*Reference*	
Living with				
Both parents	*Reference*		*Reference*	
One parent	0.87 (0.78–1.11)	0.158	0.89 (0.77–1.05) ^a^	0.164
Grandparent(s)	0.80 (0.73–0.90)	< .001	0.83 (0.76–0.90) ^a^	< .001

^a^ adjusted for children’s chronological age, BMI and family socioeconomic status (SES)

^b^ adjusted for children’s chronological age and BMI

Abbreviations: *CI* confidence interval, *PA* physical activity, *aOR* adjusted odds ratio

**Table 4:** Meeting PA recommendation by living arrangement in each subgroup^a^

Variable		Overall		Age group, years					
				9–11		12–14		15–19	
Living with		aOR (95% CI)	*p-*value	aOR (95% CI)	*p-*value	aOR (95% CI)	*p*-value	aOR (95% CI)	*p*-value
Boys	Both parents	*Reference*		*Reference*		*Reference*		*Reference*	
	One parent	0.78 (0.64–0.96)	< .050	0.78 (0.59–1.05)	0.100	0.81 (0.58–1.14)	0.226	1.11 (0.62–1.98)	0.715
	Grandparent(s)	0.79 (0.70–0.90)	< .001	0.72 (0.61–0.84)	< .001	0.94 (0.75–1.18)	0.584	1.08 (0.73–1.61)	0.683
Girls	Both parents	*Reference*		*Reference*		*Reference*		*Reference*	
	One parent	1.09 (0.86–1.40)	0.474	1.10 (0.80–1.51)	0.549	1.13 (0.72–1.79)	0.589	1.01 (0.43–2.37)	0.975
	Grandparent(s)	0.88 (0.77–0.99)	< .050	0.84 (0.72–0.98)	< .050	0.91 (0.70–1.19)	0.500	1.11 (0.59–2.09)	0.731

^a^All estimates adjusted for children’s chronological age, BMI, residence identity and family socioeconomic status (SES)

Abbreviations: *CI* confidence interval, *PA* physical activity, *aOR* adjusted odds ratio

## Discussion

Our study showed that, among 9-to-11 year old children, living with one’s grandparent(s) was associated with an increased risk of not meeting MVPA recommendations; however, this was not true among the other age groups.

The 2014 PAFSCTYS data show a low level of MVPA among school-aged children in Shanghai. Only about 18% of Shanghai school-aged children met the recommended 60 min or more of MVPA per day, and boys were more active than girls. Older children (i.e., aged 15–19 years) were less likely to meet MVPA recommendations compared to children aged 9–11 years, which was consistent with prior Chinese-based reports [28–30], studies from the United States [25, 31], and global estimates [32]. In China, adolescents in high school face the pressure of university entrance. Since academic performance is highly valued in Chinese culture, many students would study for at least 10 h per day [33]. This phenomenon has led adolescents to have less free time for necessary PA. For the difference in PA between rural and urban regions, our finding showed that children living in urban areas were less likely to meet the recommendations than those living in rural areas. While the results of national survey based on Chinese populations are somewhat inconsistent, they suggest that boys living in urban areas being more likely to meet MVPA recommendations than those living in rural areas where there may be limited availability of and access to playgrounds or PA facilities [7]. The discrepancy of the difference in children’s PA between rural and urban regions may be attributed to differences of study location. According to the survey report, at the end of 2016, public sport venues in Shanghai amounted to 1.83 m^2^ per capita, which was lower than that of other regions, and the supply-demand gap in Shanghai central areas was particularly prominent, while in rural/sub-rural areas, the supply-demand gap has been alleviated [34].

Analyses also showed family factors were associated with children’s PA. Children aged 9–11 years were more affected by living arrangement than those in other age groups. This finding was consistent with the results of previous studies. Specifically, Garcia and colleagues found that older compared with younger children were significantly less likely to receive social support for exercise [35], and Sallis and colleagues found that verbal prompts by parents were less effective for older children than for younger children [8]. This may be because parents play a more active support role when their children are younger. Positive influence from parents’ support on children’s PA level will gradually be counteracted by academic pressure [33], longer screen time [36] and some other negative influences as children grow up.

Regarding to SES influence, previous studies based in western countries have concluded that children from higher SES families usually have higher PA level [14, 37, 38]. However, present studies revealed a contrary finding that low SES level children were more likely to meet the recommendations compared to children from high SES level family. The researchers explained that, althoght children and adolescents with higher SES have higher participation in formal and organized sports, they may engage less in unstructured activites [39]. Other previous study based on Chinese populations confirmed these findings and determined that adolescents with low SES did more housework than those with high SES [40]. In addition, greater educational and economic resources increase parents’ investments in children’s intellectual and social development, which may occupy more of children’s leisure time while reducing their time and chances to engage in PA [41].

In our study, youths who lived with only one parent showed the lowest percentage of meeting MVPA recommendations. However, a few prior studies found that the inverse relation between family composition and children’s PA levels. Sallis and colleagues [42] and Wang and colleagues [19] reported that adolescents living with a single parent were more physically active than those living with both parents. They believe that children in single-parent families are cared for less-well than those living with both parents; therefore, these children have more free time and space to engage in PA [19, 42]. In addition, due to economic constraints, adolescents from single-parent families must rely on walking or biking for transportation, and usually do more housework [42]. These activities could contribute to increased PA compared to those in two-parent families [42]. Therefore, we try to observe the impact of family composition on children’s PA level after controlling the family’s socioeconomic status. As urban lifestyle changes, we believe that compared to single-parent families, two-parent families can provide youths with more time to participate in organized exercise and collective sports in urban areas of China [20].

The proportion of grandparents’ involvement in the child-rearing process is increasing due to the changing social-economic environment. Consistent with other studies, which showed a negative association between grandparents’ living habits and MVPA duration among 9-to-11 year old youths [19], participants who lived with their grandparent(s) in this study had lower odds of reaching the recommended level of PA compared with those living with their parent(s). This result may be related to the present social development in China. Grandparents taking care of grandchildren is becoming increasingly common for many dual-income families in urban areas. In fact, many children spend more time with their grandparents than with their parents [21]. Moreover, under traditional concepts, grandparents’ influence in Chinese families cannot be ignored. However, some researchers revealed that grandparents tend to overprotect their grandchildren, which might affect youths’ ability to reach the recommended level of MVPA each day [21].

This study was the first study to examine the relationship between diverse living arrangements (e.g., living with both parents, one parent, or grandparent(s)) and PA level among a large sample of youth in Shanghai, China. However, certain limitations should be noted. First, although self-report measurements demonstrate reasonable reliability [25], this measure may overestimate the PA level of children, especially in younger samples. Future research using objective methods to assess PA accurately is needed. Second, only three living arrangements were classified in the present study. Although the family structure of China is relatively consistent due to the one-child policy, it is necessary to subdivide the family composition in the future with the introduction of the two-child policy. For example, the number of siblings, the sex of siblings, and the involvement of stepparents and grandparents should be analyzed in relation to the youths’ PA. Third, although our sample comprised urban and rural youths, the results cannot be generalized to all of China, and causal conclusions cannot be inferred. Future studies should focus on other areas of China with more diverse geographical features. Last but not least, the cross-sectional design of the PAFCTYS data limit the inference to be made about the causal relationships regarding the demographic characteristics of the population and MVPA behaviors.

## Conclusion

This study revealed that less than one-fifth of school-aged children in Shanghai were meeting the daily exercise recommendations of 60 min per day. This low adherence was most evident among children aged 15–19 years and those living in urban areas as opposed to younger youth or youths living in rural areas.

Our results suggest that living arrangement plays a key role in youths’ PA, with no significant gender difference. Youths who live with one parent showed the lowest percentage of meeting MVPA recommendations compared to those living with grandparent(s) or both parents. We noted a significant living arrangement difference among children aged 9-11 years, with those living with grandparent(s) being less likely to meet MVPA recommendations compared with children living with both parents. Based on these findings, we suggest that appropriate PA promotion and intervention strategies be developed to target different subgroups (e.g., live with single parents and living with their grandparent(s)).

## Data Availability

The datasets generated and/or analyzed during the current study are not publicly available due to protect the youths’ privacy, but are available from the corresponding author on reasonable request.

## Abbreviations

aOR: Adjusted odds ratio
BMI: Body mass index
CI: Confidence interval
MVPA: Moderate-to-vigorous physical activity
OR: Odds ratio
PA: Physical activity
PAFSCTYS: The Physical Activity and Fitness in Shanghai China– The Youth Study
SES: Socioeconomic status
